# Label-Free Imaging of Basement Membranes Differentiates Normal, Precancerous, and Cancerous Colonic Tissues by Second-Harmonic Generation Microscopy

**DOI:** 10.1371/journal.pone.0038655

**Published:** 2012-06-08

**Authors:** Shuangmu Zhuo, Jun Yan, Gang Chen, Hong Shi, Xiaoqin Zhu, Jianping Lu, Jianxin Chen, Shusen Xie

**Affiliations:** 1 Institute of Laser and Optoelectronics Technology, Fujian Provincial Key Laboratory for Photonics Technology, Key Laboratory of OptoElectronic Science and Technology for Medicine of Ministry of Education, Fujian Normal University, Fuzhou, China; 2 Department of Surgery, Fujian Provincial Tumor Hospital, Fuzhou, China; 3 Department of Pathology, Fujian Provincial Tumor Hospital, Fuzhou, China; 4 Department of Endoscopy, Fujian Provincial Tumor Hospital, Fuzhou, China; University of Tokyo, Japan

## Abstract

Since changes in the basement membranes are the critical indicators for differentiating normal, precancerous, and cancerous colonic tissues, direct visualization of these warning signs is essential for the early diagnosis and treatment of colonic cancer. Here, we present that second harmonic generation (SHG) microscopy can probe the changes of basement membranes in different colonic cancer stages. Our results also show the capability of using the quantitative analyses of images for quantifying these changes in different cancer stages. These results suggest that SHG microscopy has the potential in label-freely imaging the changes of basement membranes for effectively distinguishing between normal, precancerous, and cancerous colonic tissues. To our knowledge, this is the first demonstration of the dynamics of basement membrane changes in different colonic cancer stages using entirely intrinsic source of contrast.

## Introduction

Colonic cancer is one of the major causes of morbidity and mortality in human [1]. In general, it is readily treated if diagnosed in one of the pre-invasive stages [2], [3]. However, the detection of early lesions is still a challenge. With colonic cancer development, malignant cells alter the basement membranes (interfaces of crypt and lamina propria), such as the variations in basement membrane size and density, the loss of basement membrane [4], [5]. Changes in the basement membrane are amongst the most important indicators for colonic cancer development [5], [6]. However, these warning signs have so far only been detectable by histological examination of biopsy specimens [4], [7]. Hence, the development of new and noninvasive *in situ* imaging modality for probing these warning signs is of great medical significance.

**Figure 1:**
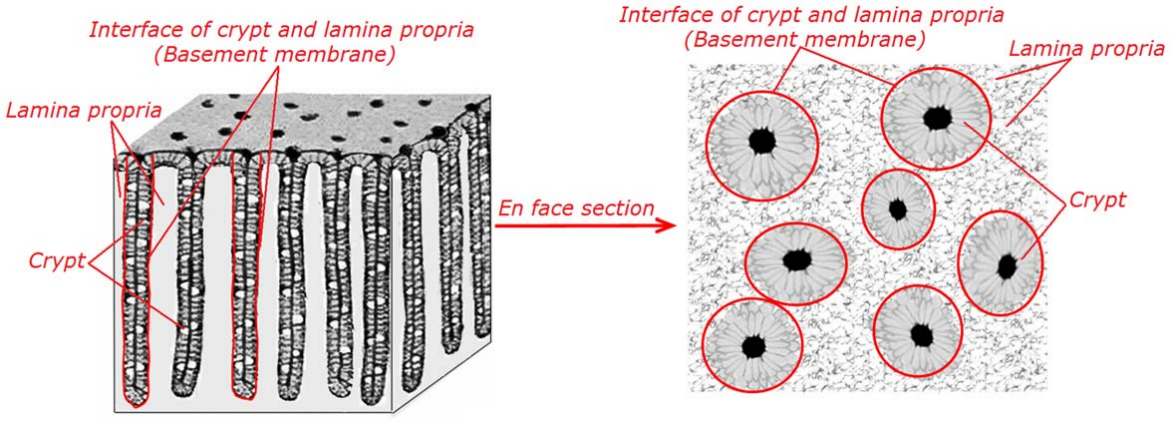
Schematic illustration of colonic mucosal structure.

Colonic mucosa contains the crypt, the basement membrane, and the lamina propria, as shown in Fig. 1. It is also seen in Fig.1 that the *en face* section of the colonic mucosa can simultaneously observe the crypt, the basement membrane, and the lamina propria. Since the crypt consists mainly of columnar epithelial cells and goblet cells that is not effective in generating second harmonic generation (SHG) signals and the lamina propria is composed primarily of collagen that is capable of emitting strong SHG signals [8]–[10], SHG microscopy may be useful for probing the changes of basement membrane that are not accessible by other imaging modalities. SHG microscopy is a nonlinear optical technique and has the advantages of being label-free, inherent three-dimension resolution, near-infrared excitation for superior optical penetration, lower photodamage, and capable of providing quantitative information [11]–[15]. Until now, there are no reports to test the potential of this technique for label-freely visualizing the basement membranes as indicators for differentiating normal, precancerous, and cancerous colonic tissues, and this is what motivated us to perform this work using this technique.

## Results

### Pathology Results

Of the 72 colonic biopsy specimens imaged, 20 were diagnosed as normal, 28 with adenoma, and 24 with adenocarcinoma. For statistical analysis, tissue samples were divided into normal case (n = 20), precancer (adenoma, n = 28), and cancer (adenocarcinoma, n = 24).

### SHG Images

**Figure 2:**
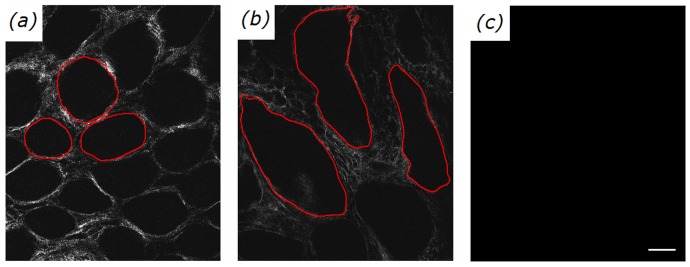
Representative SHG images from normal (a), precancerous (b), cancerous (c) colonic tissues. The excitation wavelength λ_ex_ was 800 nm. The size of images is 415×490 µm^2^ (Scale bar = 50 µm).

To probe the changes of basement membranes in different colonic cancer stages, the representative en face SHG images from normal, precancerous, and cancerous colonic tissues are respectively shown in Fig. 2. As expected, SHG imaging technique can well visualize the outline of basement membranes (red circles). It is seen in Fig. 2 that large morphological differences in the outline of the basement membranes are observed in different colonic cancer stages. In normal case, a honeycomb arrangement of round-shaped regular basement membranes with uniform size is observed. In precancer, the tubular-shaped basement membranes with larger size and a lower population density are obtained in comparison with normal case. In cancer, the basement membranes found in normal case are missing.

### Quantitative Analysis of Basement Membranes

Table 1 shows the mean and SD of the circle length of basement membrane and the population density of basement membranes for normal, precancerous, and cancerous colonic tissues. In detail, the circle length of basement membrane in normal is 342.7±27.2 µm (n = 60 areas of 20 biopsies), in precancer is 695.8±79.1 µm (n = 84 areas of 28 biopsies), and in cancer is 1767.8±166.3 µm (n = 72 areas of 24 biopsies). In addition, the population density of basement membranes in normal is 103.2±10.6/mm^2^ (n = 60 areas of 20 biopsies), in precancer is 27.1±6.3/mm^2^ (n = 84 areas of 28 biopsies), and in cancer is 0.3±1.2/mm^2^ (n = 72 areas of 24 biopsies). These results present the circle length of basement membrane and the population density of basement membranes variables which showed significant differences (*P*<0.05) between the normal, precancerous, and cancerous tissues categories based on unparired Wilcoxon rank sum tests.

**Table 1:** Quantitative variables in different colonic cancer stages.

	Circle length of basement membrane (µm)	Population density of basement membranes (mm^−2^)
Normal case	342.7±27.2	103.2±10.6
Precancer	695.8±79.1	27.1±6.3
Cancer	1767.8±166.3	0.3±1.2

## Discussion

**Figure 3:**
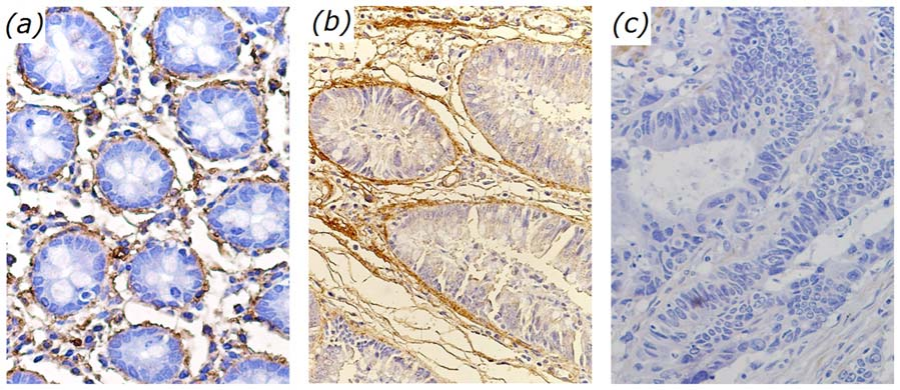
Typical images stained by primary antibodies to collagen IV from normal (a), precancerous (b), cancerous (c) colonic tissues.

This work presents that SHG microscopy can effectively probe the changes of basement membranes in different colonic cancer stages. In particular, SHG microscopy enables the identification of two morphologic features (the circle length of basement membrane, the population density of basement membranes), which show significant differences in different colonic cancer stages. These observations are consist with immunohistochemical results (shown in Fig. 3), and also confirm the fact [4], [16] that before cancer become invasive, at stages known as precancer, malignant cells alter the outline of basement membrane but do not destruct the basement membrane; as cancer become invasive, malignant cells (no SHG signals) replace collagen fibers, leading to the loss of basement membrane.

As noted, SHG microscopy is capable of label-freely imaging the changes of basement membranes in different colonic cancer stages and, as such, can act as an *in situ* histological tool that is free from the labeling requirement of conventional methods. Moreover, based on the image analyses, we were able to obtain some quantitative information in different cancer stages. Thus, SHG microscopy gives us both a morphological, as well as quantitative, tool by which to probe the changes of basement membranes in different colonic cancer stages. The important of this capability arises from the fact that the changes of basement membrane play an important role in colonic cancer development [5], [6], and these changes have so far only been detectable by histological examination of biopsy specimens [4], [7]. SHG microscopy makes it possible to observe these variations, and measuring them enables the basement membrane size and population density to be determined. These results indicate the promise of SHG imaging for clinics, and as a biomedical research tool to study the dynamics of basement membrane changes in different colonic cancer stages. Furthermore, it should be pointed out that though the inflammatory lesions were not considered in this work, SHG imaging should be able to distinguish between cancerous and inflammatory lesions due to the loss of basement membranes in cancerous tissues. In this context, one sees that SHG microscopy has the potential in label-freely visualizing the changes of basement membranes as indicators for differentiating normal, precancerous, and cancerous colonic tissues.

In conclusion, this work demonstrates the potential of SHG microscopy to label-freely image the changes of basement membranes, including the basement membrane size and population density, the important indicators of colonic cancer development. To our knowledge, this is the first time to show how SHG imaging can be used to quantitatively visualize the dynamics of basement membrane changes in different colonic cancer stages. In the future work, a large-scale study will be conducted to determine sensitivity, specificity, negative predictive value, positive predictive value with respect to traditional histological analysis. The advantage of this technique over conventional diagnostic procedures is that tissues are not required to undergo slicing, paraffin embedding, or freeze-thaw process, thus reserving the potential of *in vivo* imaging. With the capability of quantifying the basement membrane changes as shown in the present study, it is foreseeable that a SHG-based endoscopy [17]–[19] could facilitate and benefit *in vivo* studies and diagnosis in the years to come.

## Materials and Methods

### Tissue Specimen

A total of 72 fresh colonic biopsy specimens were obtained from thirty-two patients underwent endoscopic biopsy. Prior to study participation, all patients signed an informed consent, and this study was approved by Institutional Review Board of Fujian Provincial Tumor Hospital. The biopsy specimen was placed in the Glass Bottom Dish (MatTek, coverglass: 0.085–0.13 mm) for SHG imaging. In this work, the specimen preparation and SHG imaging were completed within 1 hour after endoscopic biopsy.

### Imaging Instrumentation

SHG microscopy was achieved using a nonlinear optical system which has been described previously [20]. In brief, SHG images were acquired using a commercial laser scanning microscopic imaging system (Zeiss LSM 510 META, Jena, Germany) coupled to a femtosecond Ti: sapphire laser (Coherent Mira 900-F) operating at 800 nm. The polarization direction of the laser light is the horizontal polarization. An oil immersion objective (×63 and NA = 1.4) was employed for focusing the excitation beam into tissue samples (average power less than 15 mW) and was also used to collect the backscattered intrinsic SHG signals. The images were obtained at 2.56 µs per pixel. A fine focusing stage (HRZ 200 stage, Carl Zeiss) is used to translate the samples after x-y scan of the samples for obtaining a large-area image, and to change the focus position for recording various optical sections.

### Histology Analysis

After SHG imaging, the tissue specimens were fixed in 10% formalin and prepared for pathologic examination using standard protocols. H&E-stained sections and sections stained by primary antibodies to collagen IV were obtained from each specimen, and reviewed by a certified pathologist.

### Quantification of Basement Membranes

To further quantify the changes of basement membranes in different colonic cancer stages, three 415 µm by 490 µm rectangular areas in each biopsy were selected for quantitative analysis. In this work, two analyses were performed. First, we measured the circle length of basement membrane. Moreover, to better depict this value, if there is a loss of basement membranes, the circle length of basement membrane was set to the circle length of the selected rectangular section. Second, we calculated the total number of basement membranes per unit area, that is, the population density of basement membranes. In this work, each quantitative analysis was performed on all the examined biopsies.

### Statistical Analyses

Quantitative data were summarized with the mean and standard deviation (SD), shown as “mean±SD". Summary statistics for the circle length of basement membrane and the population density of basement membranes (related to pathological determination) arise from multiple sections from each of the 72 colonic biopsy specimens taken from thirty-two patients. For the purposes of statistical analysis, the measurements from individual sections were assumed to constitute independent observations. With the data in this study (characterized by apparent homogeneity of values within the diagnostic categories), more complex statistical techniques were deemed unnecessary as they would not change the sample means, nor would they lead to larger sample standard deviations. In this work, Wilcoxon rank-sum tests were carried out to determine whether there are significant differences in the circle length of basement membrane and the population density of basement membranes between normal, precancerous, and cancerous tissues (unpaired comparison). Nonparametric Wilcoxon tests were chosen for the unpaired comparison because of the small sample size. Exact *P*-values were computed, and differences were considered to be statistically significant when the *P*-values were less than 0.05.
